# Hole Transport in A-form DNA/RNA Hybrid Duplexes

**DOI:** 10.1038/srep40293

**Published:** 2017-01-13

**Authors:** Jiun Ru Wong, Fangwei Shao

**Affiliations:** 1 Division of Chemistry and Biological Chemistry, School of Physical and Mathematical Sciences, Nanyang Technological University, 21 Nanyang Link, Singapore 637371 (Singapore)

## Abstract

DNA/RNA hybrid duplexes are prevalent in many cellular functions and are an attractive target form for electrochemical biosensing and electric nanodevice. However the electronic conductivities of DNA/RNA hybrid duplex remain relatively unexplored and limited further technological applications. Here cyclopropyl-modified deoxyribose- and ribose-adenosines were developed to explore hole transport (HT) in both DNA duplex and DNA/RNA hybrids by probing the transient hole occupancies on adenine tracts. HT yields through both B-form and A-form double helixes displayed similar shallow distance dependence, although the HT yields of DNA/RNA hybrid duplexes were lower than those of DNA duplexes. The lack of oscillatory periods and direction dependence in HT through both helixes implied efficient hole propagation can be achieved via the hole delocalization and coherent HT over adenine tracts, regardless of the structural variations.

Stacking interaction between nucleobases in DNA duplexes are of outstanding importance as they provide the necessary conditions for DNA-mediated hole transport (HT) to occur. Indeed, experimental evidences indicated that the process was capable of mediating charge up to 340 Å in a well-defined system and was finely modulated by DNA sequences and dynamics^1^,^2^. A yield compromise of DNA HT in the presence of structural interferences, such as mismatches or base lesions, emphasized the importance of maintaining the dynamical π-stack integrity during the process^3^,^4^,^5^,^6^. As such, this sensitivity becomes the basis for the development of DNA-based devices for mutagenic/SNP analysis and may be harvested within the cells by repair proteins for redox sensing and signaling across the genome^7^,^8^,^9^,^10^. Due to the dynamical nature of the DNA structure, conformational gated hopping along transiently delocalized domains over 4–5 base pairs had been proposed for the mechanism of hole propagation under various experimental conditions^11^,^12^,^13^,^14^.

Elucidation on whether hole transport could occur to the secondary structures of nucleic acids besides B-form double helix has always been of importance as it would not only evident that π-coupled orbitals of the nucleobases are the sole pathways for electron propagation, but also imply the potential roles of the process in many biological events that involve variety of complex structures composed of both deoxyribose and ribose nucleotides^15^,^16^. Among the variety of nucleic acid structures, DNA/RNA hybrids, which have A-form double helix structure formed by a pair of complementary DNA and RNA strands, are of pivotal biological significance. Beyond the well-known biological roles of DNA/RNA hybrids in DNA replication and RNA transcription of central dogma^15^, and in reverse transcription harnessed by retrovirus (e.g. HIV reverse transcriptase)^16^, recent discovery also indicated the promising therapeutic potentials of DNA/RNA hybrids in antisense therapy. With the evidence of HT as a redox chemical signaling pathway of nucleic acid binding proteins growing rapidly in the past several years^17^, to better understand HT through DNA/RNA hybrids would offer the crucial information for protein communications and expression regulations during these biological and therapeutic processes.

Both B- and A-form duplexes garner Watson-Crick base pairing and maintain the right handed antiparallel double helical structure^18^. The only chemical difference of DNA/RNA hybrids came from the C3′ endo sugar puckering in the RNA strand of the hybrid duplex due to the extra hydroxyl group on C2′. Hence merely subtle structural alternations between both duplex that would fine-tuned the dynamic motion and conformational flexibility of nucleobase pair stacks and consequently affect the electronic coupling between the flanking base pairs, while the π-coupled pathways for electron propagation in hybrid duplexes, remained similar to that in B-form duplex^19^. Hence HT in DNA/RNA hybrid duplex would allow research to focus on the effects from the conformation of base pair stacking in double helix on the electron conductivity of nucleic acids without the interference from other structural or chemical features.

HT in DNA/RNA hybrids have been explored by photochemical, biochemical, electrochemical, computational and nanotechnological approaches^18^,^19^,^20^,^21^,^22^,^23^. Early exploration of the photo-oxidative damage at GG sites and electrochemical reduction of redox intercalators in DNA monolayers on Au electrodes, showed that HT could occur in DNA/RNA hybrid duplex either in bulky solution or on metal surface, and the high sensitivity to base pair integrity also remains in DNA/RNA hybrids. However, guanine radical had a long lifetime of millisecond and HT occurring on micro to nanosecond time scale was convoluted. Thus only the static yields of the guanine damage as a net outcome after a variety of electron pathways, such as back electron transfer (BET), charge recombination, hole hopping and trapping, could be informed, which could account for the contradicting results under different experimental set-up^24^,^25^. Later the fluorescence of 2-aminopurine were used to examine the hole injection step of HT in A- and B-form duplex^19^. Although this photochemical method has advantages in high sensitivity to dynamic conformational changes of base pair stacking on nanosecond time scale, and in exploring the interstranded and intrastranded HT in both duplex structures, the critical electron propagation and trapping steps were not able to be directly revealed by HT induced fluorescence quenching of 2-aminopurine. Recently, with either the single wall carbon nanotube or scanning tunneling microscope-break junction technique^23^,^26^, electron conductivity of duplex DNA and DNA/RNA hybrids were observed on single molecular level. Despite of the small disparity on the absolute values of electronic conductivity, both techniques, like the early bulky scale experiments, confirmed the sensitive dependences of HT on the π-coupling integrity, sequences and lengths of the bridging duplexes. However, the transient hole occupancy on either DNA or RNA nucleobases during HT through DNA/RNA hybrids could not be observed by the previous studies. Whereas many unique features of DNA HT were revealed, while hole occupancy as HT yields were probed on nano- to pico-second time scales, which may avoid the interference of other electron pathways, such as BET. DNA-mediated HT were explored by using cyclopropyl-modified dG (^CP^G, ^8CP^G)^27^,^28^, dA (^CP^A)^29^ and dC (^CP^C)^30^, as the kinetic hole traps, to replace the canonical G doublets as the energetic hole traps to report the HT yeidls. Not only were these modified bases synthetically accessible and caused minimum perturbation to the integrity of DNA π-stack, the sub-nanosecond ring-opening reaction of the cyclopropyl moiety upon oxidation could also offer an ultrafast trapping rate that allow the measurement of pre-equilibrium hole delocalization during injection and propagation steps. Using cyclopropyl nucleobase substitutes allow site-specific probing of hole occupancies on the propagation pathways with minimum perturbation to the nucleic acid system and hence the direct comparison of HT efficiencies under various challenges, such as distance, bridge structures, directions of hole migration.

Herein, we report the first synthesis of cyclopropylamine modified RNA strands using cyclopropyl-substituted adenosine phosphoramidite, ^CP^a, which can probe the transient hole occupancy on RNA strand that could not be achieve before. A direct comparison of hole transport through the (dA-dT) and (dT-rA) bridge of DNA and DNA/RNA hybrid duplex, respectively, was observed. By analyzing the reactivities of ^CP^A and ^CP^a at different positions within the deoxyribose adenine or ribose adenine tract, HT efficiency in DNA and DNA/RNA duplex at pre-equilibrium state were obtained. Both of A-form and B-form duplexes exhibited similar shallow distance dependence and no HT directional preference, albeit lower photodamage yields to the hybrid duplex.

## Results

### Design of DNA and DNA/RNA hybrid duplexes

**Figure 1:**
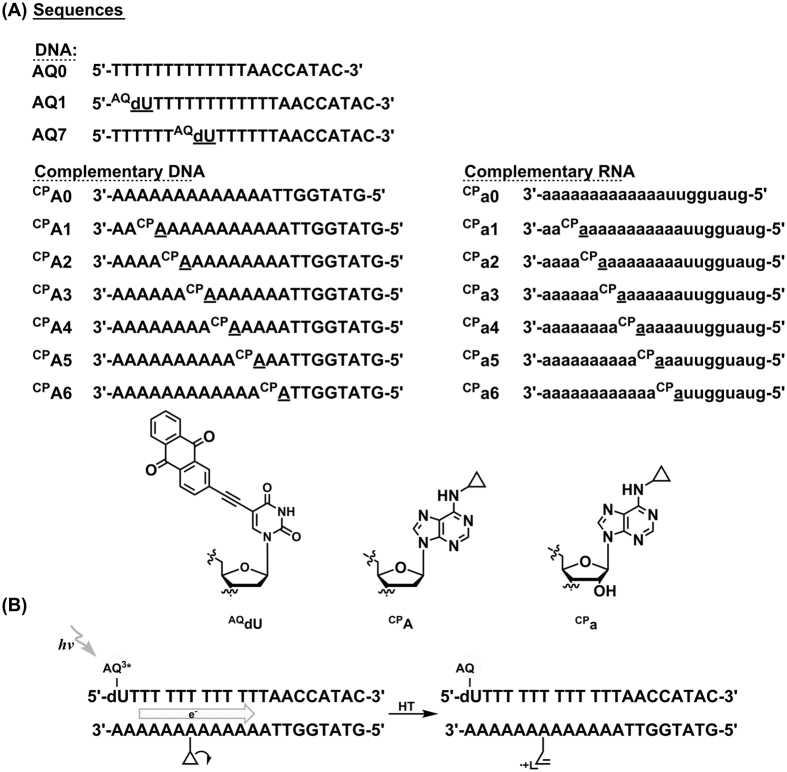
(**A**) DNA and RNA sequences (top) and structure of photo-oxidant and hole traps (bottom) utilized in this study. (**B**) Schematic illustration of the DNA-mediated HT process. Upon excitation of the photo-oxidant, a radical cation will be injected and migrate through the DNA π stack until it is trapped by the kinetic hole trap via ring opening reaction.

To explore the long range hole transport in B-form DNA and A-form DNA/RNA hybrid duplexes, a series of duplex containing photooxidant anthraquinone (AQ) and cyclopropylamine-modified deoxyribose and ribose adenine (^CP^A or ^CP^a) as hole trap were synthesized (Fig. 1A). A covalently attached AQ, via an ethynyl linker, was positioned at the 5′-terminal uridine of the thymine-rich strand, **AQ1**, to serve as the photooxidant. A series of DNA and RNA strands with ^CP^A or ^CP^a moving away from 3′-terminal of A-rich strands at two adenines per step along a 13-mer dT-dA or dT-rA bridge (^**CP**^**An** or ^**CP**^**an**, n = 1–6) were annealed to **AQ1** to form B-form and A-form duplexes, respectively. Upon irradiating AQ at 350 nm, radical-charge separation between AQ and dU on the terminal ^AQ^dU would inject a positive charge (hole) into A- or B-form adenine tract to initialize hole propagation along the duplex bridge and induce ring opening reaction once the hole reached ^CP^A or ^CP^a (Fig. 1B). The transient hole occupancy on either DNA or RNA strands could be determined by quantifying the decomposition yields of ^CP^A or ^CP^a via HPLC analysis after the enzymatic digestion of the duplex into individual nucleosides (Supplementary Fig. S1).

### Synthesis of ^CP^a-containing RNA strands (^CP^an)

**Figure 2:**
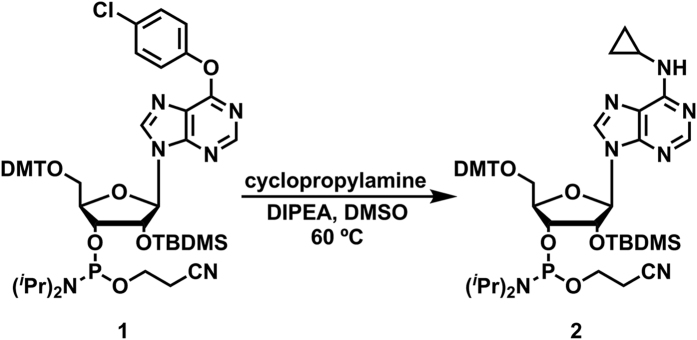
Synthesize of ^CP^a phosphoramidite (**2**) from chlorophenyl-inosine phosphoramidite via nucleophilic reaction.

The synthesis of ^CP^A-conjugated DNA strands was accomplished by post-synthetic nucleophilic reaction in aqueous cyclopropyl-amine solution^29^. However RNA oligonucleotides are vulnerable to degradation and were not stable under the post-synthetic condition, such as incubation in methanolic cyclopropylamine solution and elevated temperature (data not shown). This was presumably due to base-catalyzed silyl hydrolysis and subsequent chain cleavage; phosphodiester bonds in RNA oligonucleotides were highly susceptible to the attack from the 2′ hydroxyl group in basic solutions^31^. Hence, chemically synthesizing N^6^-cyclopropylamine-riboadenosine phosphor-amidite and site-specific incorporation into the RNA sequences via solid phase synthesis was employed instead. 2′-TBDMS-O^6^-chlorophenyl-inosine was treated with excess cyclopropylamine and DIPEA in DMSO at 60 °C overnight to install the cyclopropylamine moiety onto ribose adenosine phosphoramidite (Fig. 2). After quick extraction and chromatographic purification, ^CP^a phosphoramidite (**2**) was afforded with 90% isolated yield and verified by HRMS and ^31^P NMR (Supplementary Fig. S2). ^CP^a phosphoramidite was subsequently dissolved in anhydrous acetonitrile and incorporated into oligonucleotides without any adjustments to the standard automated RNA synthesis procedures. Standard RNA deprotection method was applied to deprotect, cleave and desilylate the RNA oligonucleotides before HPLC purifications. The intact cyclopropylamine moiety on RNA oligonucleotide was further confirmed by ESI-MS (Table S1). As shown by melting temperatures (Table S2) and CD spectra (Supplementary Fig. S3), ^CP^a was readily accommodated in A-form structures with negligible perturbation in thermal stability and helical structure, similarly as ^CP^A, the deoxyribose analog.

### One electron oxidation of ^CP^A and ^CP^a

**Figure 3:**
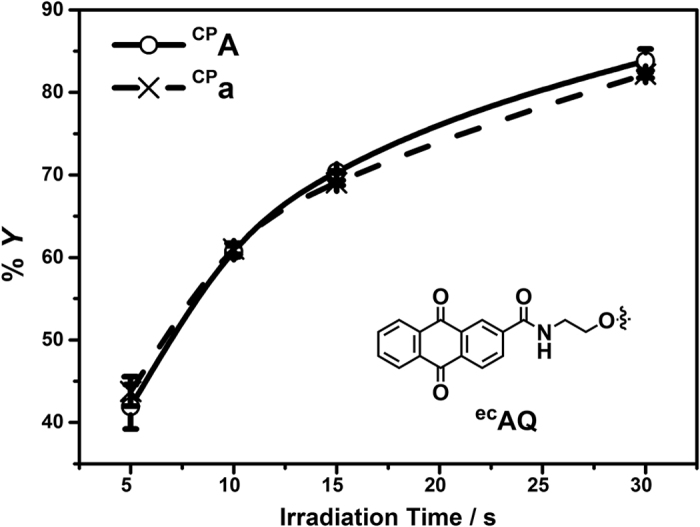
Percentage decomposition of ^CP^A of 5′-^**ec**^**AQ**^**CP**^**AT**-3′ (○) or ^CP^a of 5′-^**ec**^**AQ**^**CP**^**au**-3′ (×) as a function of irradiation time. (*insert*) Structure of ^ec^AQ as photooxidant in dinucleotides.

The efficiency of DNA HT is directly correlated with the trapping ability of the reporter. The susceptibility of ^CP^A and ^CP^a to undergo irreversible ring opening upon one-electron oxidation is of crucial importance to the accuracy of HT yields. Aside from the conformational difference arising from pentose moiety, ^CP^A and ^CP^a are structurally identical as nucleobase components. Hence it is reasonable to expect that ^CP^A and ^CP^a would not experience a significant difference in nucleobase-localized one-electron oxidation, resulting with identical ring-opening reaction. To validate our assumption that the additional 2′-OH on ^CP^a ribose would have little impact on the ring-opening reactivity of cyclopropylamine, we examined one-electron oxidation of ^CP^A and ^CP^a by photoexcited AQ in DNA and RNA dinucleotides (Fig. 3).

An end-capped AQ molecule (^ec^AQ) was tethered to the 5′-OH of ^CP^A (DNA: ^**ec**^**AQ**^**CP**^**AT**) or ^CP^a (RNA: ^**ec**^**AQ**^**CP**^**aU**) via a phosphodiester bond to facilitate direct contact and initialize a charge injection from photooxidant to trap upon irradiation (Fig. 3). The decompositions of ^CP^A and ^CP^a were then analyzed by HPLC after enzyme digestion. As anticipated, ^CP^A and ^CP^a underwent efficient ring opening reaction with nearly identical decomposition profile over the irradiation time course of 30 s. This confirmed that the rapid ring opening of the cyclopropyl moiety rendered both ^CP^A and ^CP^a comparable kinetic hole trapping processes for DNA HT, even if the base energetic might be slightly modulated by the difference in sugar composition of DNA and RNA. Therefore, ^CP^A and ^CP^a can be employed directly to measure the pre-equilibrium hole occupancy using the same trapping mechanism, while maximally maintaining the conformations of the respective DNA and RNA A-tract. Thus, any variations observed in the efficiency of ^CP^A and ^CP^a decomposition in the B-form DNA and A-form DNA/RNA duplexes would be a result of the structural influence on the base pair stacking, rather than the trapping chemistry of the two probes.

### Distance Dependence of HT in DNA and Hybrid Duplexes

To explore the distance dependence of HT in B-form DNA and A-form A-tracts, two series of duplexes, **AQ1/**^**CP**^**An** and **AQ1/**^**CP**^**an** (where n = 1–6) were used (Fig. 1A). All of the assemblies contained a ^AQ^dU as a substituent of the 5′-terminal thymine in the T-rich strands of an adenine tract with a length of thirteen AT base pairs. In the complementary adenine strands (^**CP**^**An** and ^**CP**^**an**, n = 1–6), ^CP^A or ^CP^a was serially moved away from the AQ-end with two nucleobases per step. Selective irradiation of AQ at 350 nm will lead to (1) hole injection to A-tract via oxidation of deoxyuridine by the triplet excited state of AQ; (2) hole migration through the base pair stack of adenine tract; and (3) eventually holes were trapped by ^CP^A or ^CP^a via rapid oxidative ring opening reaction of cyclopropylamine group.

**Figure 4:**
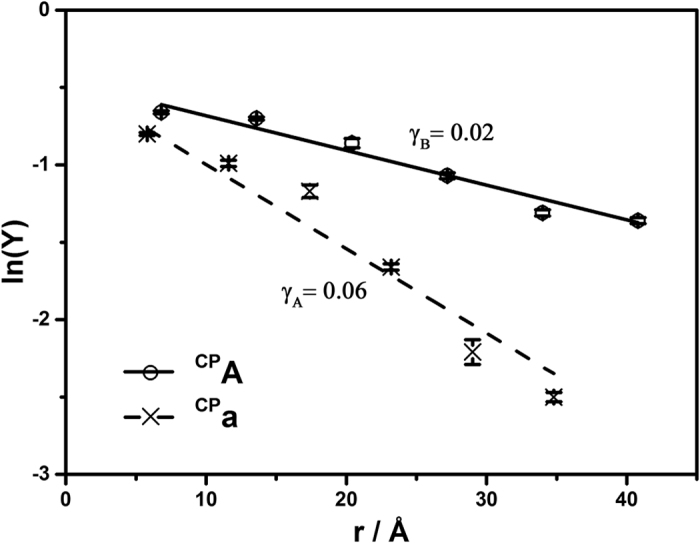
The logarithm of % decomposition of ^CP^A (•) and ^CP^a (×) (ln*Y*) as a function of bridge lengths in the duplex series of **AQ1/**^**CP**^**An** and **AQ1/**^**CP**^**an**, respectively after irradiation for 10 s at 350 nm. The distance dependences in both structures were characterized as decay parameters, γ_B_ and γ_A_ (Å^−1^) for B-form and A-form duplex, respectively. Error bars are plotted along with the data.

Supplementary Figure S4 examined the decomposition percentages of ^CP^A and ^CP^a (*Y* %) as a function of the number of bridging adenines between photooxidant and cyclopropyl trap. Efficient decomposition of both ^CP^A and ^CP^a were observed for both B-form DNA and A-form DNA/RNA duplex over 40 Å, although a slightly lower damage yield at each bridge position was observed for ^CP^a in hybrid duplex (for example, 52% for **AQ1/**^**CP**^**A1** and 45% for **AQ1/**^**CP**^**a1**). The linear fitting of the logarithmic plot of *Y* vs bridge length provided a direct analysis of the distance dependence of HT, where an average base step distance of 3.4 Å for B-form DNA and 2.9 Å for A-form hybrid DNA/RNA were taken (Fig. 4). The slope, γ_B_, obtained for B-form duplexes, **AQ1/**^**CP**^**An**, was found to be 0.02 Å^−1^. This value was comparable to the previous studies of HT across adenine tract, where the pre-equilibrium transient hole occupancy with adenine tract was reported by ^CP^A as an interior probe^32^. For instance, with Ir(III) complex as photooxidant, γ = 0.05 Å^−1^ were obtained over a 9 base pairs of A-tract^33^. In the case of hybrid duplex series, **AQ1/**^**CP**^**an**, the decay parameter of hole transport over distance was obtained as γ_A_ = 0.06 Å^−1^. The shallow distance dependence for A-form double helix was comparable to that of B-form duplex, despite a lower oxidative damage to adenine via photoinduced HT. Remarkably, even though HT in hybrid duplex was initialized with distinct methods, both features of distance dependence as a larger γ_A_ for hybrid duplex and a small γ_A_ less than 0.1 for hybrid adenine tract was also observed by single molecular measurements with STM technique^23^. The similar γ values for both double helical structures suggested that the HT mechanisms in A- and B-form duplexes may share common features while mediating hole delocalization over adenine tracts, which was not unexpected due to the fact that only subtle variation on the dynamic motion and stacking structure of the base pairs occurred between the two types of duplexes. In addition to the shallow dependence on bridge length, no apparent oscillatory periods on HT yields were observed, indicating that step-wise hopping mechanism with small domains over adjacent base pairs as the hole carriers may not be dominant in hybrid duplex, similar as in B-form adenine tracts^34^.

### Direction Dependence of Hole Transport in DNA and Hybrid Duplexes

The directional dependence of DNA-mediated HT in both B-form DNA and A-form DNA/RNA hybrid duplexes was further examined with the duplex series of **AQ7**. Anthraquinone was positioned in the center thymine in the T-rich strand (**AQ7**) to allow charge migration towards both the 3′- and 5′- ends of the duplexes simultaneously (Fig. 1A). Two complementary DNA and RNA strands were selected to report HT yields of both propagation directions, ^**CP**^**A1** and ^**CP**^**a1** for 5′ → 3′ HT, and ^**CP**^**A5**, ^**CP**^**a5** for 3′ → 5′ HT. The numbers of AT base pairs on the bridge for both directions were the same, while the relative orientation of hole traps to the photooxidant were placed opposite in the two sets of the duplexes.

**Figure 5:**
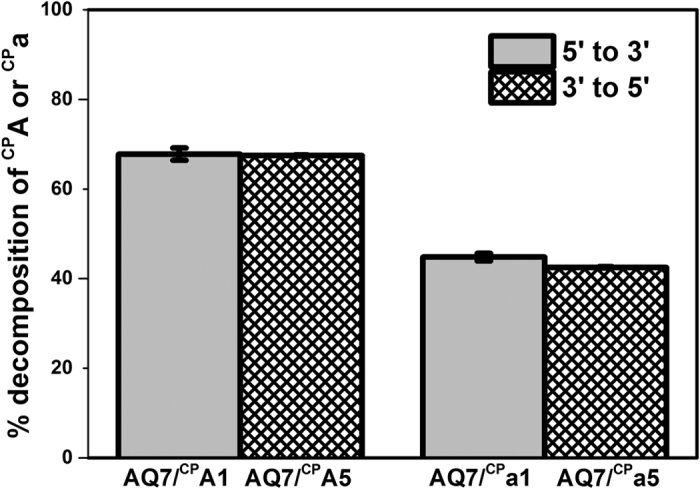
% Decomposition of ^CP^A in **AQ7/**^**CP**^**A1**, **AQ7/**^**CP**^**A5** and ^CP^a in **AQ7/**^**CP**^**a1**, **AQ7/**^**CP**^**a5** after irradiation for 10 s at 350 nm. Error bars are plotted along with the data.

As depicted in Fig. 5, ^CP^A in both **AQ7/**^**CP**^**A1** and **AQ7/**^**CP**^**A5** showed the same decomposition yields. The same situation was observed in the hybrid duplex, although the yields of HT observed here was still lower than that of DNA duplex. There was no significant difference in HT yields between both directions of electron propagation, regardless of the secondary structures of the duplexes. Neither the B-form nor the A-form duplexes showed any preference in the specific direction of hole migration.

## Discussion

Efficient HT over duplex DNA or DNA/RNA hybrid was observed to induce the decomposition of both ^CP^A and ^CP^a over 40 Å. The linear decay of logarithmic HT yields according to the bridge length showed no oscillatory pattern. The lack of periodicity and efficient HT in both structures indicated that rA-dT tracts in A-form hybrid duplexes, similarly as dA-dT tracts in B-form DNA duplex, can accommodate fast coherent HT by a delocalized HT domain over the entire A-tract^25^. Furthermore, the lack of direction dependence supported the idea that a fully delocalized domain can form over the entire adenine tract, regardless of the pentose backbone. Once a positive charge was injected into a duplex π-stack, the radical cation would rapidly delocalize into single coherent domain on A-tract or a-tract equally via both HT directions due to the isoenergetics of adenine tracts. As shown in Fig. 5, no direction difference was observed for HT in both DNA duplex and RNA/DNA hybrids. This indicated that a large domain delocalized over at least 7 adenine base pairs could form on both A-form and B-form adenine tracts to achieve efficient coherent HT. Although A-form holds a set of helical parameters slightly different from B-form double helix, which may account for the lower HT efficiencies, A-form DNA/RNA hybrid duplex was also structurally and dynamically capable of forming an extensive delocalized domain across the adenine bridge. The consistent features observed in both DNA and DNA/RNA helixes indicated that hole transport through duplex adenine tract could undergo the same coherent HT via delocalizing domains. The features as directional and distance dependence are not a function of one particular DNA structures but is instead a universal characteristic feature of HT over duplex nucleic acids with optimized sequences.

## Conclusion

In summary, cyclopropyl-adenine had been successfully synthesized as ribose phosphoramidite and incorporated into RNA oligonucleotides. Oxidative ring opening reaction of both ^CP^A and ^CP^a in a fixed length of DNA and RNA A-tract allowed us to characterize the distance and directional dependence of HT in both B-form DNA and A-form DNA/RNA duplexes at a fast time scale. Our results clearly suggested that both types of double helical secondary structures showed the similar shallow distance dependence with no obvious oscillatory pattern, while RNA/DNA hybrids were less susceptible to the long-range photooxidation than DNA duplexes with the same base sequence context. Significantly, there was no directional preference for hole migration towards 5′ or 3′ end for both the B-form and A-form duplexes. The above data, taken together, indicated that extensive delocalization of positive charges over adenine bases was independent on the identity of helical structures involved. Our findings have noteworthy implications that nucleic acids in central dogma upon folding into various secondary structures will exhibit different resistance against the harmful UV irradiation, while a consistent HT mechanism may be employed by chemical signaling pathways through nucleic acids.

## Methods

### Synthesis of 5′-Dimethoxytrityl-N^6^-cyclopropylinosine, 2′-O-TBDMS-3′-[(2-cyanoethyl)-(N, N-diisopropyl)]-phosphoramidite (2)

In a microtube, 5′-dimethoxytrityl-O^6^-chlorophenyl, 2′-O-TBDMS-3′-[(2-cyanoethyl)-(N, N-diisopropyl)]-phosphoramidite (**1**, 100 mg, 0.1 mmol) was allowed to react with cyclopropylamine (57 mg, 1 mmol) and diisopropylethylamine (51 mg, 0.5 mmol) in DMSO (100 *μ*L). The reaction was carried out in a heating block at 60 °C for 18 h or until TLC showed complete elimination of the starting material. Water (200 *μ*L) was added to the reaction mixture and the crude product was extracted repeatedly with ethyl acetate and dried over MgSO_4_. The solution was concentrated under pressure and applied to a silica column (pre-equilibrated with 1% TEA) using hexane/ethyl acetate (3:1) as the eluent to give **2** as a white foam (83 mg, 90%). TLC (H:EA, 1:1 v/v): R_f_ = 0.81; ^31^P NMR (400 MHz, CDCl_3_): δ = 148.9, 150.9 ppm; HRMS (ESI) (m/z): [M + H]^+^ calcd for C_49_H_67_N_7_O_7_PSi, 924.4609; found, 924.4596.

### Oligonucleotides Synthesis

Cyanoethyl phosphoramidite of O^6^-chlorophenyl-I and anthraquinone-5-ethynl-dU were purchased from Berry and Associates. All trityl-on DNA and RNA sequences were prepared on Bioautomation Mermade 4 DNA/RNA synthesizer following standard phosphoramidite protocols using chemicals and CPG (1 mmol) from Glen Research. The oligonucleotides were then cleaved off from the solid support and deprotected by treating with ammonium hydroxide/methylamine mixture (v/v, 1:1) at 65 °C for 10 min. After preparation, the DNA and RNA solutions were concentrated to dryness. Following, the TBDMS-protected RNA strands were desilyated using triethylamine trihydrofluoride (TEA.3HF) in DMSO and TEA. The DNA and RNA oligonucleotides were purified by reverse-phase HPLC (microsorb 100–5 C18 Dynamax column, 250 mm× 10.0 mm) with a triethylammonium acetate/acetonitrile gradient (5–30% of acetonitrile over 30 min). After lyophilization, the oligonucleotides were treated with 80% glacial acetic acid and repurified with reverse-phase HPLC. The identities of the desired DNA and RNA strands were confirmed by ESI-mass spectrometry and quantified by UV-Vis spectroscopy. ^CP^A- and ^ec^AQ-tethered strands were prepared according to previous reports, respectively^29^,^35^.

### Photooxidant Experiments

Aliquots (10 *μ*M, 30 *μ*L) in 20 mM sodium phosphate buffer, 100 mM NaCl, pH 7.0 were prepared by annealing the ^AQ^dU-DNA conjugates with its ^CP^A-DNA or ^CP^A-RNA complements (ratio of 1.1:1) at 90 °C for 5 min and gradually cooling to room temperature overnight. The samples were irradiated at 350 nm with a 450 W Xenon lamp equipped with a monochromator and a 320 nm long pass filter. To analyze for ^CP^A or ^CP^a decomposition following irradiation, the samples were digested completely into free nucleosides with phosphodiesterase I and alkaline phosphatase at 37 °C for 24 h. The digested nucleosides were analyzed by reverse-phase HPLC (Chemcobond 5-ODS-H column, 4.6 mm × 150 mm) with a triethylammonium acetate/acetonitrile eluent. The decomposition yield of ^CP^A or ^CP^a was taken as the difference between the ratio of their HPLC peak areas in an irradiated sample over that of dark control, with thymidine as the internal standard. All photo-oxidations were carried out at least three times and the results were averaged with the errors expressed as standard deviation.

## Additional Information

**How to cite this article:** Wong, J. R. and Shao, F. Hole Transport in A-form DNA/RNA Hybrid Duplexes. *Sci. Rep.* **7**, 40293; doi: 10.1038/srep40293 (2017).

**Publisher's note:** Springer Nature remains neutral with regard to jurisdictional claims in published maps and institutional affiliations.

## Supplementary Material

Supporting Documents
