# Supplementary material for: Hole Transport in A-form DNA/RNA Hybrid Duplexes
Source: Sci Rep. 2017 Jan 13;7:40293. doi: 10.1038/srep40293 (PMC5233965; doi:10.1038/srep40293)
Supplement: Supporting Documents [file srep40293-s1.pdf]

Supporting information for:

Hole Transport in A-form DNA/RNA Hybrid Duplexes

Jiun Ru Wong[1] and Fangwei Shao[1*]

[1]Division of Chemistry and Biological Chemistry, School of Physical and Mathematical Sciences, Nanyang Technological University, 21 Nanyang Link, Singapore 637371 (Singapore)

*fwshao@ntu.edu.sg

(a)

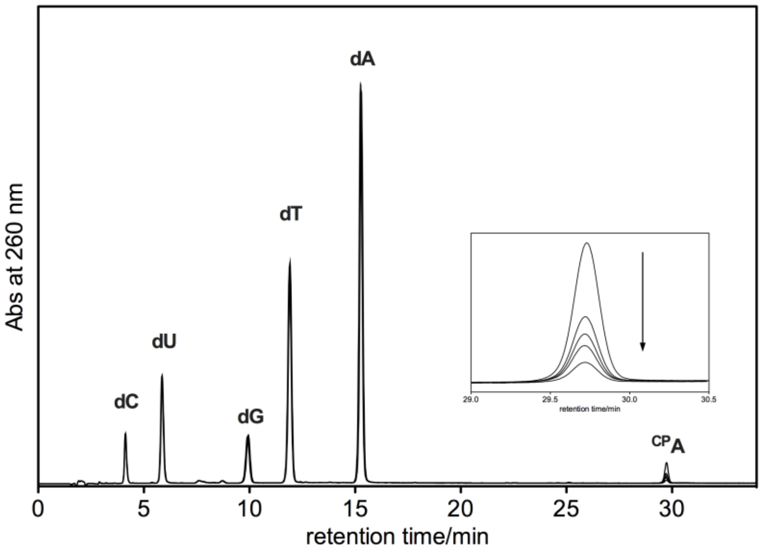

(b)

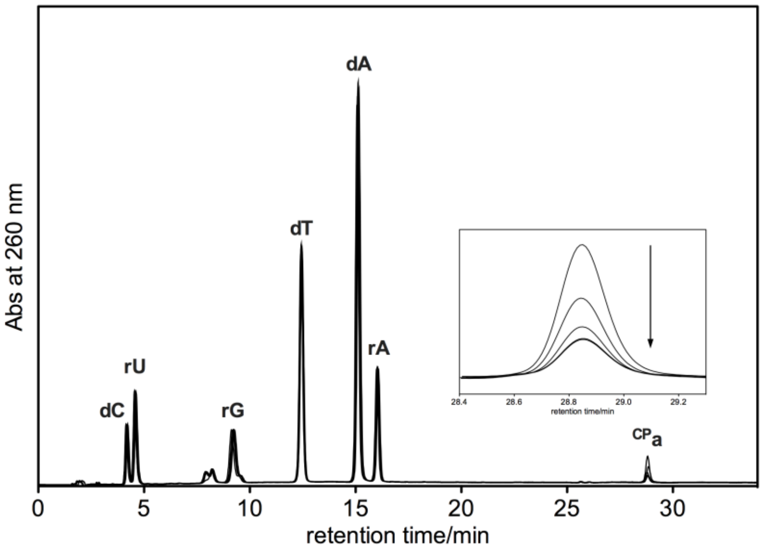

FIGURE S1. Overlaid HPLC profiles for digested nucleosides from (a) **AQ1^{CP}A1** and (b) **AQ1^{CP}a1** after irradiation at 350 nm for 0, 10, 30, 60 and 180s. Insert: decomposition of ^{CP}A or ^{CP}a with increasing irradiation time. dU was added to duplex DNA samples as external standard for HPLC analysis.

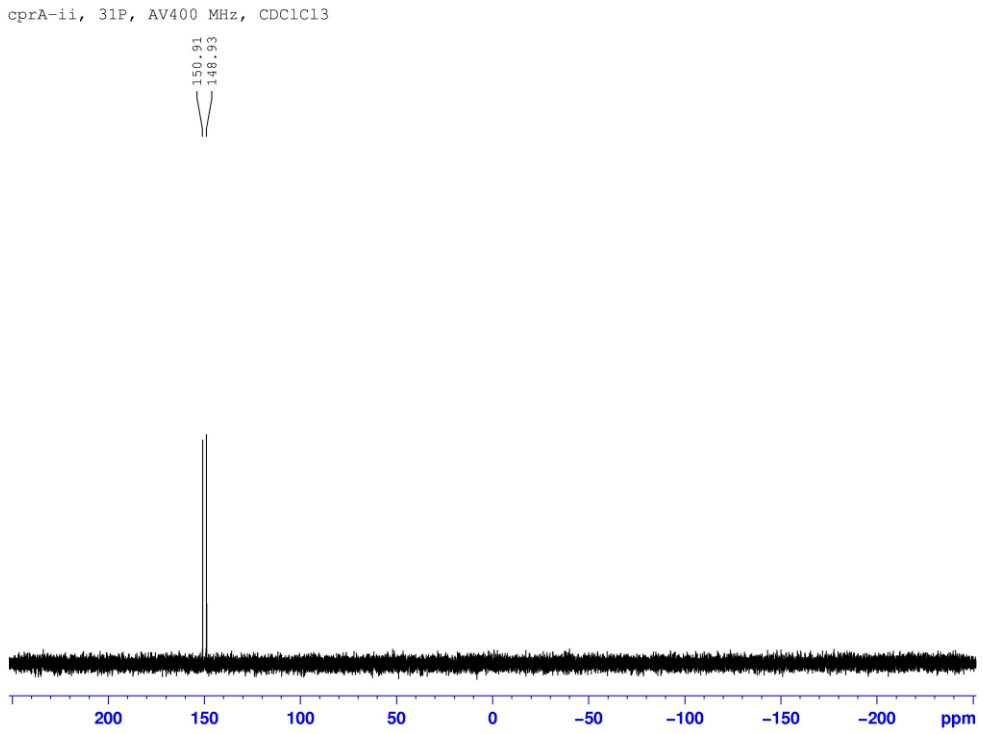

FIGURE S2. ^{31}P NMR of ^{CP}a phosphoramidite (2).

TABLE S1. ESI-MS characterisation for DNA and RNA oligonucleotides.

DNA or RNA	ESI-mass calc. [M+H]$^+$	ESI-mass found [M+H]$^+$
AQ1	6533.2	6533.7
AQ7	6533.2	6536.0
^{CP}A1	6567.0	6567.2
^{CP}A2	6567.0	6567.7
^{CP}A3	6567.0	6567.2
^{CP}A4	6567.0	6569.4
^{CP}A5	6567.0	6569.6
^{CP}A6	6567.0	6567.3
^{CP}a0	6807.2	6807.9
^{CP}a1	6847.3	6847.3
^{CP}a2	6847.3	6847.5
^{CP}a3	6847.3	6847.7
^{CP}a4	6847.3	6847.5
^{CP}a5	6847.3	6847.4
^{CP}a6	6847.3	6848.1

Thermal Analysis. The thermal denaturation profile (T_m) were measured using a Shimadzu UV-2550 with temperature control attachment. The absorbance of 1.5 µM duplexes (20 mM sodium phosphate buffer, 100 mM NaCl, pH 7.0) was monitored every 1 ºC at 260 nm from 15 ºC to 90 ºC with a heating rate of 0.5 ºC/min. The data was fitted to a sigmodal curve to determine the T_m. The error of T_m (expressed as standard deviation) was obtained over at least three sets of individual measurements.

TABLE S2. Melting temperature (T_m) of DNA and DNA/RNA duplexes [a]

Duplex	AQ0	AQ1	AQ7
^{CP}A0	49.8 (±0.1)	50.1 (±0.2)	
^{CP}A1	47.8 (±0.1)	48.2 (±0.1)	43.0 (±0.1)
^{CP}A5			42.3 (±0.2)
^{CP}a0	45.2 (±0.2)	46.2 (±0.2)	
^{CP}a1	41.3 (±0.2)	42.4 (±0.2)	35.1 (±0.0)
^{CP}a5			34.7 (±0.2)

[a] T_m was averaged over three data sets and the standard derivation was showed in bracket. Analysis was performed with 1.5 µM of duplexes in 20 mM sodium phosphate buffer (pH 7.0), 100 mM NaCl.

Circular Dichroism. The same duplexes from the melting experiments were used for CD analysis. All CD spectra were collected on a JASCO-18 CD instrument at room temperature, at a rate of 100 nm/min and wavelength increment of 0.1 nm. The spectra obtained were an average of ten sequential scans corrected for ellipticity (mdegrees) readings obtained from the buffer (20 mM sodium phosphate buffer, 100 mM NaCl, pH 7.0).

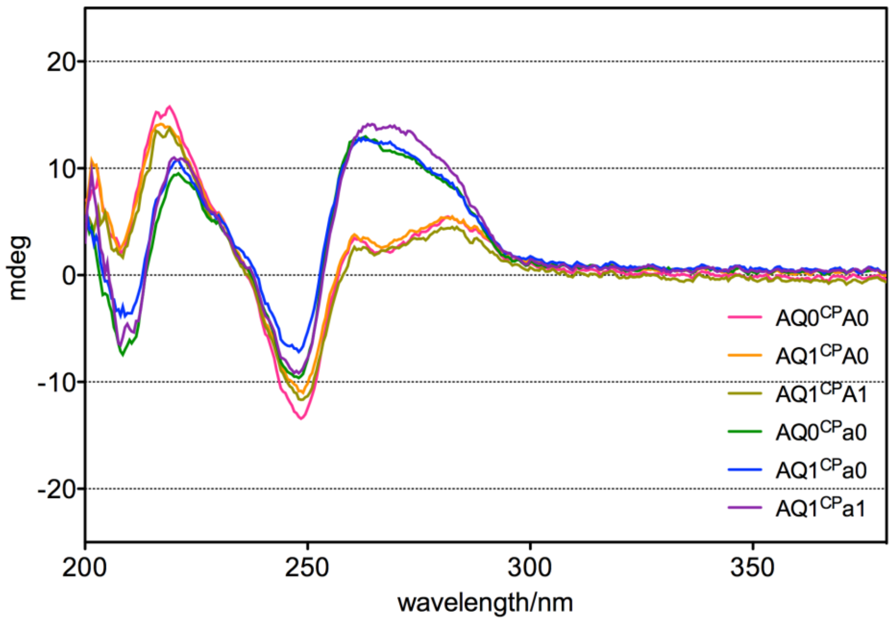

FIGURE S3. CD spectra of various B-form and A-form hybrid duplexes. Analysis was performed with 1.5 µM of duplexes in 20 mM sodium phosphate buffer (pH 7.0), 100 mM NaCl.

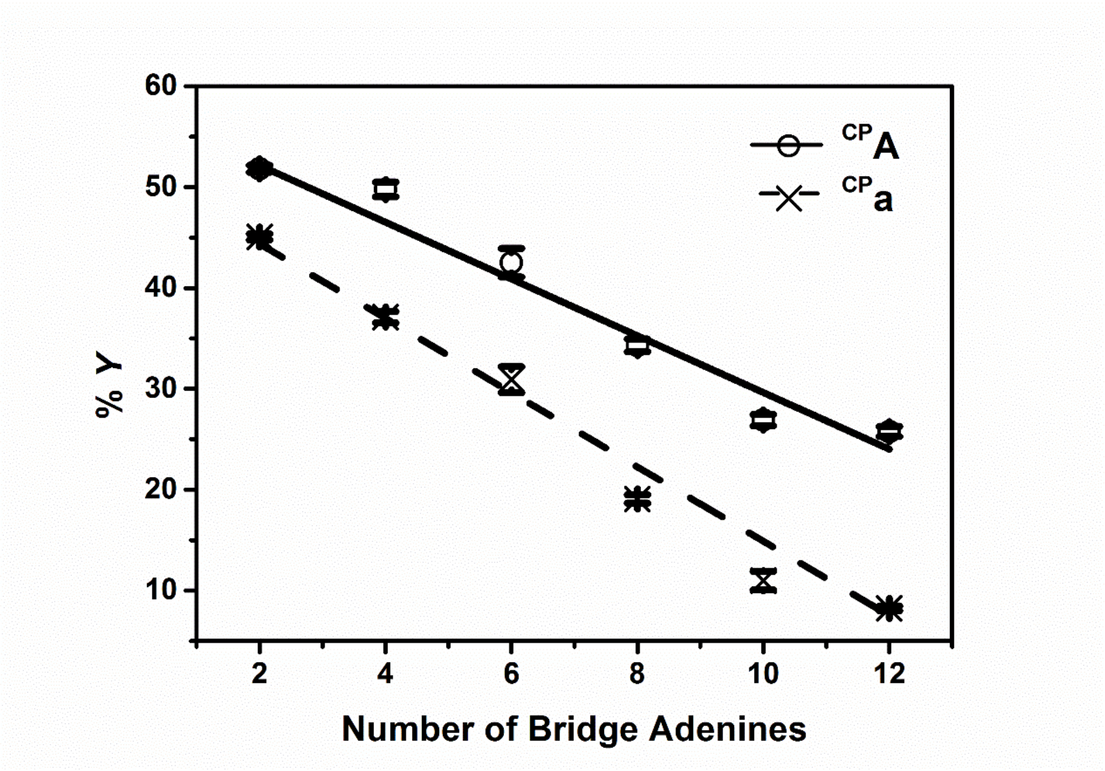

FIGURE S4. % Decomposition of ^{CP}A (○) and ^{CP}a (×) (% *Y*) as a function of bridge position for **AQ1/^{CP}An** and **AQ1/^{CP}an** respectively after irradiation for 10 s at 350 nm. Error bars are plotted along with the data spots.
